# Investigation of the effect of Jacobson’s relaxation technique on the fatigue of family caregivers of hemodialysis patients: a single-blinded randomized controlled trial

**DOI:** 10.1186/s40001-024-01641-w

**Published:** 2024-01-11

**Authors:** Seyedeh Azam Sajadi, Farzaneh Ravash, Zahra Farsi

**Affiliations:** 1 Department of Nursing Management, School of Nursing, Aja University of Medical Sciences, Tehran, Iran; 2 Critical Care Department, School of Nursing, Aja University of Medical Sciences, Tehran, Iran; 3 Research and Community Health Departments, School of Nursing, Aja University of Medical Sciences, Tehran, Iran

**Keywords:** Relaxation therapy, Fatigue, Dialysis, Caregivers, Family

## Abstract

**Background:**

Caring for hemodialysis patients could be a heavy burden on family caregivers, causing them to become fatigued and decrease their quality of life. This study aimed to investigate whether Jacobson’s relaxation can help alleviate the fatigue of family caregivers of hemodialysis patients.

**Methods:**

This randomized controlled trial was conducted in 2021. Sixty-six family caregivers of hemodialysis patients were recruited by convenience sampling from a referral hospital in Tehran, Iran, and assigned randomly by coin toss to two groups of experimental (*n* = 32) and control (*n* = 34). Caregivers in the experimental group performed Jacobson’s relaxation three times a week, each time for 30–45 min, for 30 days. The score and severity of fatigue before, 2 weeks after, and 1 month after the intervention were measured with the Fatigue Severity Scale. Data analysis was performed in the statistics software SPSS using descriptive statistics (frequency, percentage, mean, and standard deviation) and analytic statistics (Independent Samples *t*-test, Mann–Whitney *U* test, Chi-Square test, Fisher’s exact test, and RM-ANOVA test). The significance level was less than 0.05.

**Results:**

The fatigue scores of the experimental and control groups were not significantly different before the intervention (4.42 ± 0.42 vs. 4.38 ± 0.42, *P* = 0.696). However, the experimental group had significantly lower fatigue scores than the control group 2 weeks after the intervention (4.11 ± 0.63 vs. 4.39 ± 0.42, *P* = 0.036) and 1 month after the intervention (3.5 ± 0.71 vs. 4.4 ± 0.44, *P* = 0.001). The results also showed a significant drop in the fatigue score of the experimental group after the intervention (*P* < 0.0001), but no such change in the control group (*P* = 0.662).

**Conclusion:**

Jacobson’s relaxation technique was effective in alleviating the fatigue of family caregivers of hemodialysis patients. Nurses are therefore recommended to promote the technique as a safe and easy method of fatigue management for family caregivers.

## Introduction

Chronic kidney disease is a global health problem that imposes a heavy burden on patients and their family members [1]. The global prevalence of chronic renal failure (CRF) is 242 cases per 1,000,000 people and is increasing by 8% each year [2]. In Iran, this rate is about 12% per year, which is 4% higher than the global average [3].

Hemodialysis is the most common kidney replacement therapy for these patients [4]. Patients undergoing hemodialysis face special problems such as the need for regular hemodialysis sessions, following a complex diet, taking multiple medications, complications related to vascular access, and the need for frequent visits, diagnostic tests, and frequent hospitalizations in hospitals. In this situation, apart from the patient, the person who is most affected by the disease and the treatment process is the family caregiver [5].

Family members play an essential role in managing the disease and improving the quality of life of CRF patients undergoing hemodialysis [6]. In Iran, there are usually strong emotional ties and a sense of commitment between family members. Family members are among the most important sources of support for patients [1]. The supportive role for caregivers of hemodialysis patients may put them under a lot of pressure [7]. Family caregivers are forgotten saviors, bearing not only the physical caregiving responsibilities but also the emotional burdens of caring for a family member with a debilitating disease [8].

A good understanding of the conditions of these caregivers can help the healthcare system give them the psychological support they need to continue caring for dialysis patients. Reducing the care burden of family caregivers can lead to the improvement of their quality of life (QoL) and general health, which will have benefits such as increased survival, adherence to treatment, and QoL for patients with CRF who undergo hemodialysis [5, 9, 10].

One of the major problems for caregivers of patients with CRF is the feeling of excessive fatigue [11]. Fatigue is defined as the lack of physical and mental energy or feeling of tiredness, perceived by the individual or caregiver, that interferes with the usual and desired activities of daily life [12]. Research has shown that 59–69% of caregivers of hemodialysis patients suffer from fatigue due to their caregiving duties [11, 13]. There is also evidence that the family caregivers of hemodialysis patients experience stress, anxiety, depression, financial and communication issues, low adaptability and QoL, high caregiving pressure, loss of sleep quality, and a lot of fatigue [9, 14].

There are a wide variety of non-pharmacological interventions for alleviating fatigue, including music therapy, yoga, energy therapy [9], aromatherapy, massage therapy, cognitive-behavioral methods, acupressure, and relaxation [15]. Also, since pharmacological methods can be costly and may have negative side effects on various organs, they are rarely used as the first line of treatment. Thus, it is increasingly common to use complementary and non-pharmacological methods of fatigue management [9, 16, 17], which are highly regarded for being inexpensive, non-invasive, simple to implement, and having no chemical side effects [17].

One complementary method is relaxation. Jacobson’s relaxation technique is a relaxation method introduced in 1970 that is more popular than alternatives because it is easier to teach and learn [18]. As a non-pharmacological intervention with the ability to learn easily, relaxation can be effective in reducing stress and tension [19]. Jacobson’s relaxation method involves tightening and relaxing muscles for a few seconds in sequence. Progressive muscle relaxation originates from the theory that psychological biological conditions, including neuromuscular pressure, are the basis of many unpleasant emotional feelings and physical and mental illnesses. Muscle relaxation leads to relaxation of the mind because an emotional state will not exist in the presence of complete relaxation of the body parts. Relaxation prevents the generation of negative thoughts and emotions such as anxiety and tension and neutralizes the effects of increased muscle tension on the body [20].

There is evidence that Jacobson’s relaxation technique alleviates a wide range of physical and psychological symptoms such as anxiety, pain, depression, poor mood and self-confidence, and stress. For example, Nazari et al. reported that performing Jacobson’s relaxation technique for 40 min twice a week for 4 weeks was effective in reducing fatigue and pain intensity in patients with multiple sclerosis [21]. Sahin et al. also reported that the technique was effective in reducing fatigue and improving the sleep quality of patients with chronic obstructive pulmonary disease [22].

The hemodialysis process can have profound impacts on the lives of not only the patients but also their family members. However, most studies in this field focus on the patient, ignoring the issues of family caregivers. From the perspective of holistic nursing, it is crucial to pay attention to all aspects of the health of not only patients but also their family members. Since the authors’ search in credible databases revealed no finding regarding the effect of Jacobson’s relaxation on the fatigue of family caregivers of hemodialysis patients, this study aimed to investigate whether this relaxation method can help alleviate the fatigue of these caregivers.

## Materials and methods

### Study design

This randomized controlled trial was conducted in 2021. It was registered on the Iranian Registry of Clinical Trials (NO: IRCT20210808052114N1, registration date: 14/08/2021).

### Participants and sampling

Seventy family caregivers of hemodialysis patients were recruited using the convenience sampling method from the people attending a referral hospital in Tehran, Iran. The caregivers were randomly allocated by coin toss into two groups: experimental (scheduled to meet on even days of the week) and control (scheduled to meet on odd days of the week). The sample size, 32 per group, was estimated based on the relative frequency of fatigue in the study of Dehkordi et al. [23] at a 95% confidence level and an 80% test power (Eq. 1). However, 35 caregivers were recruited for each group to account for the possibility of a 10% dropout rate.

1 $${\text{n}} = \frac{{\left( {z_{{1 - \frac{\alpha }{2}}} + z_{{1 - \beta }} } \right)^{2} \left[ {p_{1} \left( {1 - p_{1} } \right) + p_{2} \left( {1 - p_{2} } \right)} \right]}}{{\left( {p_{1} - p_{2} } \right)^{2} }};n = \frac{{\left( {1.96 + 0.86} \right)^{2} \left[ {0.15(0.85) + 0.45(0.55)} \right]}}{{(0.15 - 0.45)^{2} }} = 31.8$$

The inclusion criteria were: age of 18 to 60 years, willingness to participate in the study, ability to read and write in Persian, being the patient’s family member, being the patient’s main caregiver for at least 6 months, and the patient performing three rounds of hemodialysis a week. The exclusion criteria were: withdrawal from the study, not attending the initial training session, and not performing at least 10 of the 12 relaxation sessions.

### Data collection

Before the intervention, the intensity of fatigue in both groups was measured. Data collection was performed using an individual characteristics questionnaire (age, gender, marital status, education level, employment status, relationship with the patient, income level, hours of care provided per day, daily exercise, and patient’s dialysis history) and the Fatigue Severity Scale (FSS). FSS, which was developed in 1989 by Krupp et al., measures people’s perceptions of their fatigue on nine items scored from one to seven on a Likert scale. The FSS’s total score is divided by nine, giving a final score ranging from one (no fatigue) to seven (maximum fatigue). A score of less than two indicates low fatigue; a score between two and four indicates moderate fatigue; and a score of five and above indicates severe fatigue [9]. The validity and reliability of this tool have been confirmed in Iranian studies. For example, the reliability of the Persian version of FSS has been established with an intraclass correlation coefficient (ICC) of 0.93 and Cronbach’s alpha of 0.96 [24]. In this study, the Cronbach’s alpha was o.71.

### Intervention

Before the study, one researcher, who is a master’s student in nursing with 10 years of clinical work experience in different hospital departments, completed a training course on relaxation techniques under the supervision of a psychiatric specialist. During the study, this researcher taught Jacobson’s relaxation technique to each caregiver of the experimental group individually in a 30-to-45-min face-to-face session held in the hospital’s companion resting room. In the same session, the researcher asked the caregivers to perform the technique once (to see whether they had learned it) and instructed them to do the relaxation technique three times a week [25] for 4 weeks [21] at home or in the resting room when attending the hospital for their patient’s hemodialysis. The researcher also gave these caregivers a checklist for recording the date and duration of their relaxation sessions or the reason they had not performed them. Jacobson’s relaxation technique was taught in these stages: (1) explaining the steps of the relaxation technique, (2) explaining about the muscles by which the technique is performed, (3) the researcher performing the progressive muscle relaxation technique, and (4) answering caregivers’ questions and asking them to perform the technique. The follow-up was performed by making phone calls to caregivers once a week and visiting them when they were attending the hospital to ensure they used the relaxation technique.

**Fig. 1:**
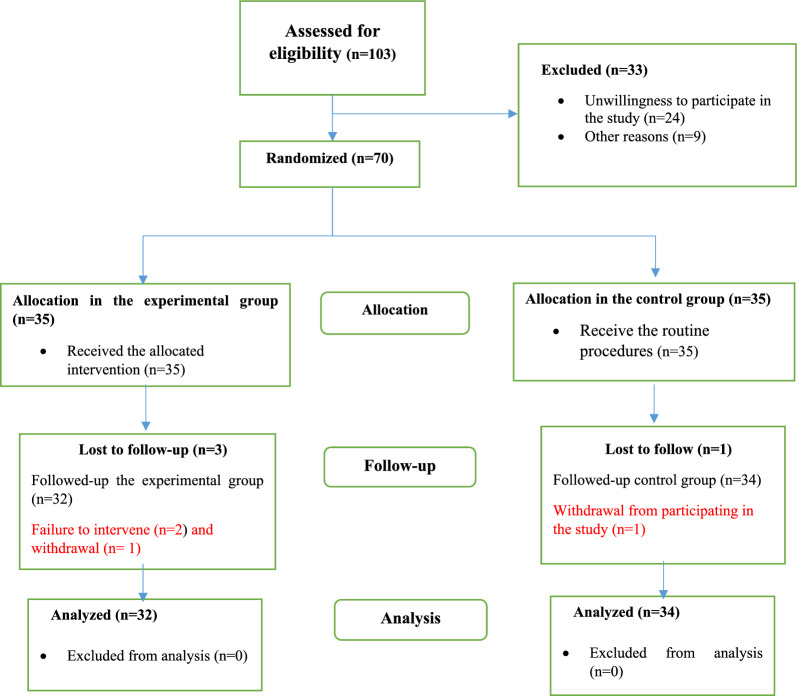
Process of the study

The fatigue of all caregivers in both groups was measured again 2 weeks and 4 weeks after the start of the intervention. The process of the study is illustrated in Fig. 1.

### Data analysis

Data analysis was performed in the statistics software SPSS using descriptive statistics (frequency, percentage, mean, and standard deviation) and analytic statistics (independent Samples *t*-test, Chi-Square test, Fisher’s exact test, and RM-ANOVA). The Kolmogorov–Simonov was used to check the normality of the data distribution. The independent samples *t*-test and Mann–Whitney *U* test were used to compare quantitative demographic variables. The Fisher’s exact test and Chi-square test were used to compare categorized variables. The independent samples *t*-test was used to compare the fatigue score between the two groups before, 2 and 4 weeks after the intervention. The RM-ANOVA was used to show the interaction effect of time and intervention. This test compared the fatigue score at baseline, 2 and 4 weeks after the intervention between two groups. The significance level in all statistical tests was less than 0.05. The statistical analyst was blinded to group allocations.

### Ethical considerations.

The study was approved by the Ethics Committee of Aja University of Medical Sciences (IR.AJAUMS.REC.1399.263). Informed oral and written consent was obtained from all patients. The researcher explained the study objectives to all caregivers and asked them to sign the informed consent form approved by the ethics committee of the university. The researchers conducted the study in compliance with the research ethics principles in the Declaration of Helsinki and guaranteed that the information of all participating caregivers would remain confidential. The researchers also properly informed caregivers that participation in the study is voluntary and does not affect the treatment and care provided to their patients, and they may withdraw from the study at any time should they wish to do so. They also tried to adhere to the principles of the Committee on Publication Ethics in relation to publication ethics.

## Results

During the study, three people from the experimental group were excluded because of non-participation in the intervention and withdrawal, and one person from the control group was excluded because of withdrawal, leaving 66 caregivers in the study (32 in the experimental group and 34 in the control group).

The caregivers had a mean age of 48.93 ± 1.67 years and were mostly women (77.3%), in the age range of 51–60 years (48.49%), housewives (69.70%), with high school education (74.74%), and were not doing any sports activity (84.85%). Most caregivers were the wives of the patients (75.75%), who were providing all the care the patients needed round the clock. There were no significant differences between the two groups in terms of patients’ and caregivers’ age, gender, number of children, education level, patients’ history of hemodialysis, caregivers’ occupation, relationship with patients, daily exercise, or hours of care provided (*P* > 0.05) (Tables 1, 2).

**Table 1:** Individual characteristics (Quantitative Variables) of family caregivers of hemodialysis patients

Quantitative Variables	Experimental group (mean ± SD)	Control group (mean ± SD)	Test results	*P*-value
Caregiver’s age (years)	48.15 ± 13.06	49.67 ± 12.67	Mann–Whitney *U* Test Z =− 0.54	0.59
Patient’s age (years)	56.58 ± 5.52	58.58 ± 4.61	Independent Samples *t –*Test t =− 1.37	0.17
Number of children	2.03 ± 0.99	2.06 ± .085	Mann – Whitney *U* Test Z = − 0.34	0.87
History of hemodialysis (years)	4.15 ± 1.81	4.38 ± 1.55	Independent Samples *t –*Test t = − 0.54	0.58

SD: Standard deviation; f: Frequency

**Table 2:** Individual characteristics (categorized variables) of family caregivers of hemodialysis patients

categorized Variables		Experimental group f (%)	Control group f (%)	Test results *P*-value
Caregiver’s gender	Male	9 (28.1)	6 (17.6)	Fisher’s exact test *P* = 0.38
	Female	23 (71.9)	28 (82.4)	
Patient’s gender	Male	22 (31.3)	27 (79.4)	Fisher’s exact test *P* = 0.40
	Female	10 (68.8)	7(20.6)	
Caregiver’s education level	Junior-high-school diploma	4 (12.5)	3(8.80	Chi-Square test X^2^ = 0.37 *P* = 0.92
	High-school diploma	23 (71.9)	26 (76.5)	
	Academic degree	5 (15.6)	5 (14.7)	
Patient’s education level	Illiterate	10 (31.3)	11 (32.4)	Chi-Square test X^2^ = 3.39 *P* = 0.32
	Junior-high-school	10 (31.3)	16 (47.1)	
	Diploma	6 (18.8)	5 (14.7)	
	Academic degree	6 (18.8)	2 (5.9)	
Occupation	Housewife	20 (62.5)	26 (76.5)	Chi-Square test X^2^ = 1.74 *P* = 0.69
	Employed	7 (21.9)	5 (14.7)	
	Unemployed	2 (6.3)	1 (2.9)	
	Retired	3 (9.4)	2 (5.9)	
Kinship	Spouse	24 (75)	26 (76.5)	Chi-Square test X^2^ = 0.41 *P* = 0.91
	offspring	6 (18.8)	5 (14.7)	
	others	2 (6.2)	3 (8.8)	
Hours of daily care provided	Up to eight hours	3 (9.4)	2 (5.9)	Chi-Square test X^2^ = 0.40 *P* = 0.92
	up to 12 h	6 (18.8)	7 (20.6)	
	in total	23 (71.9)	25 (73.5)	
Daily exercise	Yes	6 (18.8)	4 (11.8)	Fisher’s exact test *P* = 0.50
	No	26 (81.2)	30 (88.2)	

f: Frequency

The independent sample *t*-test showed no significant difference between the two groups in terms of fatigue severity before the intervention (*P* = 0.696). However, the independent sample *t*-test showed significantly lower fatigue in the experimental group than in the control group 2 weeks (*P* = 0.036) and 1 month after the intervention (*P* = 0.001) (Table 3).

**Table 3:** Comparison fatigue score of family caregivers of hemodialysis patients before and after the intervention

Stage	Pre-test				Post-test				Follow up				RM-ANOVA
Fatigue score	Experimental group		Control group		Experimental group		Control group		Experimental group		Control group		
	Mean	SD	Mean	SD	Mean	SD	Mean	SD	Mean	SD	Mean	SD	
	4.42	0.43	4.38	0.42	4.11	0.63	4.39	0.42	3.5	0.71	4.4	0.44	
Independent Samples- *t* Test	*t* = 0.393				*t* = − 2.17				*t* = − 5.75				F = 70.182
	df = 64				df = 64				df = 64				df = 2
	*P* = 0.696				*P* = 0.036				*P* = 0.001				*P* < 0.0001

**Fig. 2:**
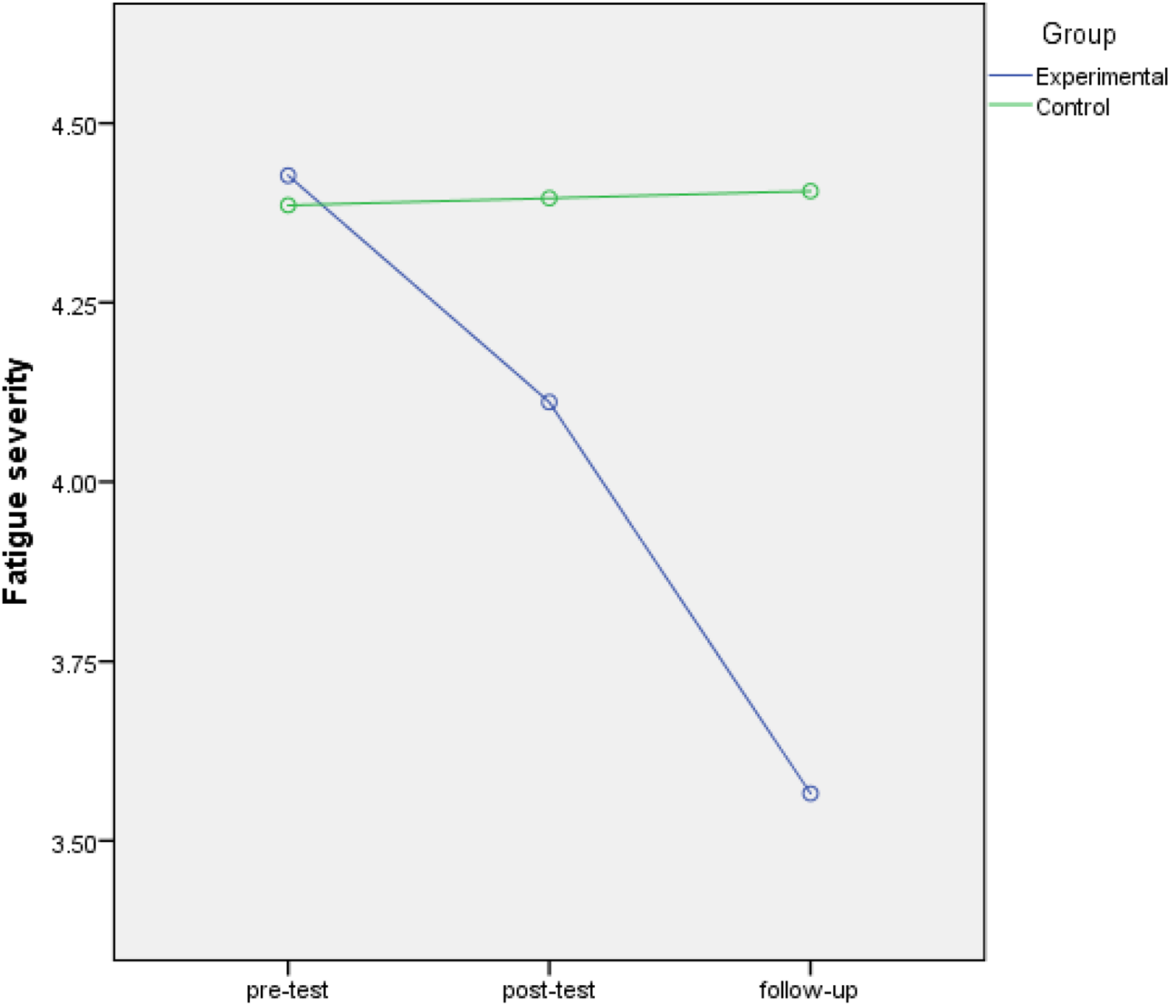
Changes in fatigue score in the experimental and control groups over time

The RM-ANOVA test showed a significant drop in the fatigue of caregivers in the experimental group after the intervention (F = 76.3, df = 2, *P* < 0.0001), but no significant change in this respect in the control group (F = 0.415, df = 2, *P* = 0.662) (Table 3). The interaction effect of time and Jacobson’s relaxation technique on the fatigue scores of caregivers in the two groups are compared in Fig. 2 and Table 3.

Based on Partial Eta Squared, the overall effect sizes of Jacobson’s relaxation technique on the fatigue scores of caregivers were 0.98.

The findings showed that 90.9% of caregivers had severe fatigue before the intervention, but this ratio decreased to 68.2% 2 weeks after the intervention and 54.5% 1 month after the intervention (Table 4).

**Table 4:** Fatigue severity of family caregivers of hemodialysis patients before and after the intervention

Stage	Fatigue severity	Experimental	Control	Total
		f (%)	f (%)	f (%)
Pre-test	Moderate	2 (6.3)	4 (11.8)	6 (9.1)
	Severe	30 (93.8)	30 (88.2)	60 (90.9)
Test, *P*-value	Fisher’s exact test, *P* = 0.673			
Post-test	Moderate	17 (53.1)	4 (11.8)	21 (31.8)
	Severe	15 (46.9)	30 (88.2)	45 (68.2)
Test, *P*-value	Fisher’s exact test, *P* < 0.0001			
Follow-up	Moderate	26 (81.3)	4 (11.8)	30 (45.5)
	Severe	6 (18.8)	30 (88.2)	36 (54.5)
Test, *P*-value	Fisher’s exact test, *P* < 0.0001			

f: Frequency

## Discussion

This study aimed to determine whether Jacobson’s relaxation technique would be effective in alleviating the fatigue of family caregivers of hemodialysis patients. In this study, the mean fatigue scores of caregivers in the experimental and control groups were not significantly different before the intervention, but the fatigue of the experimental group decreased significantly 2 weeks and 1 month after the intervention. Findings showed that the overall effect size of Jacobson’s relaxation technique on fatigue of family caregivers of hemodialysis patients was high. Therefore, it can be concluded that Jacobson’s relaxation technique is effective in reducing the severity of caregivers’ fatigue. This finding is not unusual. Because the relaxation of the body is accompanied by the release of hormones and chemical substances; therefore, this process causes physical changes, like increased blood flow in the hands and feet, and reduces the severity of fatigue [26].

This conclusion follows several previous reports. For example, Pouraboli et al. reported that using relaxation techniques was significantly effective in reducing the fatigue and anxiety of parents who were family caregivers of children with leukemia undergoing chemotherapy [27]. In a study by Choi, music therapy, progressive muscle relaxation, and the combination of the two techniques were all found to have positive impacts on the anxiety, fatigue, and QoL of family caregivers of hospice patients [28]. In a study conducted to determine the effect of progressive muscle relaxation on fatigue among 72 pre-hospital emergency staff in the Medical Emergency and Accident Management Center, the results indicated a significant reduction in fatigue after the intervention [29]. Although none of the above studies were conducted on family caregivers of hemodialysis patients, relaxation intervention was implemented on informal and formal caregivers in all of them.

There is also evidence that relaxation alleviates fatigue in patients with multiple sclerosis [15, 26]. Another study has also shown that muscle relaxation is effective in reducing the fatigue of hemodialysis patients [30]. In another study, it was shown that progressive muscle relaxation led to reduced fatigue in a population of Iranian elderly [23]. The authors found no study recorded in credible databases whose results would contravene the above findings. In most of the previous studies, the effect of relaxation on patients’ or caregivers' burden was investigated, which indicates the innovation and strength of the present study. Another strength of the study was the follow-up of caregiver fatigue 2 and 4 weeks after the intervention.

One limitation of this study was the indirect monitoring of adherence to the intervention since the researchers chose not to visit participants at their homes. However, one researcher tried to make sure of adherence by making regular phone calls to caregivers and visiting them when they were attending the hospital’s hemodialysis department.

## Conclusion

The findings showed that Jacobson’s relaxation technique was effective in alleviating the fatigue of family caregivers of hemodialysis patients. This technique can therefore be recommended as a simple, free, easy, and effective non-pharmacological intervention for fatigue management in these caregivers. Since family caregivers are unfamiliar with such methods, hemodialysis and public health nurses can reduce these caregivers' fatigue by teaching them how to relax with these techniques. It is also recommended to investigate the effect of relaxation on other physical and mental problems of family caregivers of hemodialysis patients and other chronic patients, including diabetes and cancer.

## Data Availability

The datasets used and analyzed during the present study are available from the corresponding author on reasonable request.

## Abbreviations

FSS: Fatigue severity scale
CRF: Chronic renal failure
QoL: Quality of life
